# Older Adults’ Experience of Health and Life Events as Reported Online Over 150 Consecutive Weeks

**DOI:** 10.1093/geroni/igaa057.645

**Published:** 2020-12-16

**Authors:** Jeffrey Kaye, Nora Mattek, Zachary Beattie, Nicole Sharma, Thomas Riley, Hiroko Dodge

**Affiliations:** 1 Layton Alzheimer’s Disease Center, Portland, Oregon, United States; 2 Oregon Health & Science University, Portland, Oregon, United States; 3 Pregon Health & Science University, Portland, Oregon, United States

## Abstract

Key activities and life events that impact quality of life and health may be gleaned from cross-sectional annual surveys or for medical data, derived intermittently from electronic health records. These methods cannot reflect the day-to-day and week-to-week changes that typically occur in domestic life. To assess the frequency and types of major life activities and events occurring at finer-grained temporal scale, we queried online, every week, three domains of activity and function (life events such as having an overnight visitor, travel away from home; health-related events such as medication changes, falls, ER/hospital visits, health limitations; and internal states such as pain, mood, or loneliness). Over a mean assessment period of 2.9 ± 1.2 years, 16,738 online surveys were completed by 129 community-residing volunteers with mean age 84 years. Overall the most frequent events reported were physical health limitations (14%), travel away from home (12%) and overnight visitors (9%). Needing new in-home assistance for medication management, bathing, dressing or grooming was rare (1%). Accidents were also rare (1%). Low mood, pain ratings (0-10 scale) and loneliness were infrequently reported; 4% low mood, 8% pain rated > 5, 3% reporting loneliness. Low mood and increased pain intensity were highly correlated with health changes (medication changes, ER/hospital visits, health limitations, falls; all p<0.0001). Continuous home-based online assessment of life activities and health can provide a more detailed and timely characterization of older adult function. These data may be used to guide more timely and effective health maintenance programs and interventions.

